# Metastatic Prostate Cancer—A Review of Current Treatment Options and Promising New Approaches

**DOI:** 10.3390/cancers15020461

**Published:** 2023-01-11

**Authors:** Philip Posdzich, Christopher Darr, Thomas Hilser, Milan Wahl, Ken Herrmann, Boris Hadaschik, Viktor Grünwald

**Affiliations:** 1 Department of Urology, University Hospital Essen, 45147 Essen, Germany; 2 Department of Internal Medicine (Oncology), University Hospital Essen, 45147 Essen, Germany; 3 Department of Nuclear Medicine, University Hospital Essen, 45147 Essen, Germany

**Keywords:** prostate cancer, ADT, PSMA Bite, PSMA radioligand therapy, immunotherapy, bispecific T-cell engager

## Abstract

**Simple Summary:**

Prostate cancer is the most common tumor in men. Although there have been many new developments in the last few years, metastatic castration resistant prostate cancer remains a deadly disease. This article provides an overview of currently approved treatment options as well as new therapies that are not standard of care yet. All relevant developments from classical androgen deprivation therapy (ADT) to bispecific T-cell engagers (BiTE) are considered.

**Abstract:**

Androgen deprivation therapy (ADT) alone has been the standard of care for many years in men with metastatic prostate cancer. Due to the limited survival under this monotherapy, many new treatment options have been developed in the last few years. Regarding hormone-sensitive prostate cancer, combination therapies of two or three agents of ADT, androgen receptor signaling inhibitors (ARSI) and chemotherapy have been established and led to a significant benefit in overall survival. Additionally, in patients with metastatic castration-resistant prostate cancer, there are many new therapeutic approaches. Chemotherapy alone has been the standard of care in this situation. In the last years, some new therapeutic options have been developed, which led to an improved survival after progression under chemotherapy. These therapies include ARSI, PARP inhibitors and Lu-PSMA radioligand therapy. The use of a bispecific T-cell engager (BiTE) in this setting is a new promising therapeutic approach, which has not been established as standard of care yet. The role of immunotherapy in prostate cancer is still under investigation. Overall, many new treatment options make prostate cancer therapy a challenging and promising field.

## 1. Introduction

Prostate cancer is the most common solid cancer in men worldwide. The number of estimated new prostate cancers in the US in 2022 was 268,490. The number of estimated deaths due to prostate cancer is 34,500 [1]. Surgery and radiotherapy are the mainstay of treatment in localized disease. Androgen-deprivation-therapy (ADT), androgen signaling inhibition (ARSI) and chemotherapy dominate the standard medical treatment in recurrent or metastatic disease. However, the majority of patients acquire castration-resistance, which is associated with a poor prognosis.

The latest advancements in systemic treatments were the addition of Poly ADP-Ribose Polymerase Inhibitor (PARPi) and radio-ligand therapies (RLT) to the armentarium. While check point inhibitors (CPI) have revolutionized anti-cancer treatment in many diseases they were less successful in prostate cancer. A major driver of this immune resistance is derived from the dominance of a non-inflamed “cold” tumor environment in prostate cancer [2]. However, some activity has been noted for ipilimumab in men with mCRPC who were treated with at least one chemotherapy and had evidence of progression after the discontinuation of ADT [3], as well as for pembrolizumab in a subgroup of genetically instable prostate cancer patients [4].

Novel concepts are needed to advance immunotherapies in prostate cancer. Sipuleucel-T indicated that T-cell-based therapies were clinically active in principle and have led to further clinical development. Bispecific T Cell engager is a novel promising anti-cancer treatment modality in non-inflamed cancers, such as prostate cancer. These compounds re-direct T-cells to the tumor environment by targeting a cancer-specific epitope, such as PSMA in prostate cancer, which is linked to a component of the T-cell receptor (TCR). This mechanism recruits T-cells to the tumor milieu by binding to prostate cancer cells, which activates T-cells and enables immunologic anti-tumor response. In this article, we will give an overview of the current therapy standard in mHSPC and mCRPC, and introduce bispecific T-cell-engager as a new mechanism of action to overcome immune resistance.

## 2. Methods

A review of the Medline database through PubMed was conducted to identify pivotal trials of approved and contemporary medical treatments in prostate cancer. Guideline-recommended regimens were selected and the current EAU Guideline on prostate cancer served as a reference.

## 3. Current Treatment Options in Metastatic Prostate Cancer

### 3.1. Metastatic Hormone-Sensitive Prostate Cancer (mHSPC)

#### 3.1.1. Prognostic Factors

Several criteria have been established to estimate the prognosis of metastatic hormone-sensitive prostate cancer. Most common criteria are CHAARTED and LATITUDE criteria. These are summarized in Table 1 and Table 2.

The occurrence of “de novo” metastatic hormone-sensitive disease at the time of initial diagnosis was identified as a poor prognostic factor. The median overall survival (OS) was 51.6 months in low volume disease (HR = 1.64; 95% CI 1.16–2.31) and 43.2 months in high volume disease (HR = 2.48, 95% CI 1.83–3.36) compared to the reference group (prior local treatment/low volume disease HR = 1). These results differed substantially from those patients who had local therapy prior to the diagnosis of metastatic disease, i.e., metachronous metastatic prostate cancer. In this population, OS in low volume disease was 92.4 months (HR = 1) and 55.2 months in high volume disease (HR = 1.9, 95% CI 1.31–2.75 [7].

#### 3.1.2. ADT

ADT is the standard treatment approach in mHSPC patients and may consist of Luteinizing hormone-releasing hormone (LHRH) agonists, LHRH antagonists or bilateral orchiectomy. In previous decades, ADT resided as single modality, but this is not the standard of care (SOC) anymore. OS in mHSPC patients treated with ADT alone is about 42 months [8].

Today, therapies that combine ADT with ARSI, chemotherapy, or both, yielded superior OS results. Single agent ADT has limited value and remains an option in elderly, frail patients, only.

#### 3.1.3. Combination Therapies

The advent of combination therapies has intensified medical treatment and improved OS prognosis in prostate cancer patients with mHSPC. Different components were added to the ADT backbone.

An early set of treatment intensification trials tested the combination of ADT + docetaxel. There were two important trials, which investigated the combination of ADT and Docetaxel.

The STAMPEDE trial included patients with newly diagnosed M1 or N+ disease, locally advanced disease (cT3, cT4, ISUP grade at least 4, PSA at least 40 ng/mL) and patients with relapse after local treatment (PSA at least 4 ng/mL or PSA doubling time under 6 months or PSA level over 20 ng/mL, M or N relapse). In all, 689/724 (95%) of patients in the control arm and 347/362 patients in the Docetaxel arm had no previous treatment. A total of 1184 patients received the standard of care (ADT) and 592 patients received ADT + docetaxel. The median OS in the ADT group was 43.1 months, the estimated 5-year OS was 37% (CI 34–41%). In contrast, the median OS in patients who received ADT + Docetaxel was 59.1 months, the estimated 5-year OS was 49% (*p* = 0.003, HR = 0.81, 95% CI 0.69–0.95) [9].

The second Important trial was called CHAARTED, which also investigated the impact of ADT + docetaxel in patients with mHSPC and ECOG performance score 0–2. A distinction was made between high volume and low volume disease. In the overall patient population, OS was 57.6 months in the ADT + docetaxel group in contrast to 47.2 months in the ADT alone group (HR = 0.72, 95% CI 0.59 to 0.89; *p* = 0.0018). Subgroups of patients with high and low volume disease achieved different treatment effects. While ADT + docetaxel vs. ADT achieved a superior OS of 51.2 vs. 34.4 months (HR = 0.63, 95% CI 0.50 to 0.79; *p* < 0.001) in high volume disease, there was no benefit in patients with low volume disease in longterm follow-up (HR = 1.04, 95% CI 0.70 to 1.55; *p* = 0.86) [10].

As a result, OS in newly diagnosed metastatic hormone-sensitive prostate cancer is superior for ADT + docetaxel than ADT alone, but that effect of chemotherapy is restricted to high volume disease only.

#### 3.1.4. ADT + Androgen Receptor Signaling Inhibitors (ARSI)

##### Abiraterone Acetate: Selective Inhibitor of Steroid 17α-Hydroxylase (CYP17A1)

In this section, we will discuss the impact of three different androgen receptor signaling inhibitors in addition to ADT.

The STAMPEDE trial also investigated the impact of Abiraterone Acetate (AA), a selective inhibitor of the enzyme steroid 17α-hydroxylase (CYP17A1) and prednisone in addition to ADT. Inclusion criteria were already mentioned above. In all, 957 received ADT alone, 960 patients were treated with ADT and Abiratrone Acetate + prednisone (AAP). There was a significant benefit in OS (HR = 0.63, 95% CI 0.52 to 0.76; *p* < 0.001) in patients who received the combination therapy which corresponded to 3-year OS rates 83% vs. 76%. Metastatic status at time of randomization had no significant impact on treatment effect (*p* = 0.37) [11].

In the LATITUDE trial, ADT + Placebo (*n* = 597 pts.) was compared to ADT + AAP (*n* = 602 pts.). Only patients with high-risk newly diagnosed metastatic disease, ISUP grade > 4, at least three bone lesions or measurable visceral metastases were included. The combination of ADT + AAP reported significant OS improvement (HR = 0.62, 95% CI 0.51–0.76; *p* < 0.001) when compared to ADT alone. Three year OS rates were: 66% in ADT + AAP vs. 49% in ADT + Placebo group [6].

In summary, the combination therapy of ADT + AAP provided a significant survival benefit over ADT monotherapy.

##### Enzalutamide: A Competitive Androgen Receptor Blocker

The ENZAMET trial included mHSPC patients (with distant metastases (M1) and ECOG score 0–2). 1125 men (588 with high volume disease, 537 with low volume disease) were 1:1 randomized to receive enzalutamide + ADT or ADT + standard nonsteroidal antiandrogen drug (bicalutamide, nilutamide, flutamide). Overall survival favored ADT + enzalutamide (HR = 0.67, 95% CI 0.52–0.86; *p* = 0.002) and 3 year OS rate were 80% in the Enzalutamide group and 72% for ADT alone. Regarding the volume of disease, the proportion alive after 36 months was 0.82 (0.75 to 0.87) in the control group vs. 0.90 (0.84 to 0.93) in the Enzalutamide group and low volume disease. In high volume disease, proportion alive after 36 months was 0.64 (0.58 to 0.70) in the control group and 0.71 (0.64 to 0.76) in the Enzalutamide group. There was no statistically significant impact of volume of disease (*p* = 0.14) [12].

##### Apalutamide: Inhibitor of the Ligand-Binding Domain of the Androgen Receptor

In the TITAN study, apalutamide + ADT (*n* = 525) was compared to ADT + Placebo (*n* = 527) in patients with mHSPC. A total of 94 of 525 patients in the Apalutamide group and 79 of 527 in the placebo group had previous treatment for localized disease. The other patients were de novo metastasized. The study reported superior OS in favor for the combination arm (HR = 0.67, 95% CI 0.51–0.89; *p* = 0.005). After 24 months, there was an overall survival of 82.4% in the Apalutamide group, but only 73.5% OS in the Placebo group [13] In the final analysis after 405 deaths, it was shown that apalutamide decreased the risk of death by 35% (HR = 0.65; 95% CI, 0.53 to 0.79; *p* < 0.0001) [14].

The results of these trials indicated that the combination therapy of ADT and ARSI improved OS in mHSPC patients than ADT alone. Both patient groups, high volume disease and low volume disease had a benefit from combination therapy.

#### 3.1.5. Triple Combinations (ADT + ARSI + Docetaxel)

In this section, triple combinations in the treatment of mHSPC will be discussed.

Darolutamide is a competitive androgen receptor inhibitor and reported improved metastasis free survival and overall survival in non-metastatic CRPC patients. The principal activity paved the way for further testing in an earlier setting. The ARASENS trial investigated the combination of darolutamide + ADT + docetaxel (*n* = 651) compared to placebo + ADT + docetaxel (*n* = 655). The primary analysis at first data cut-off showed that the risk of death was reduced by 32.5% in the triple-therapy arm when compared to the ADT + docetaxel doublet [15].

PEACE-1 is a complex study and consisted of four arms. At this point, only the combination therapy of ADT + docetaxel with or without abiraterone acetate and prednisone (AAP) were considered for analyses. The median rPFS was prolonged by 2.5 years in patients who received the AAP containing triplet (HR = 0.5, 95% CI 0.40–0.62; *p* < 0.0001). Furthermore, there was a 25% reduction in the risk of death in patients who received the triplet (HR = 0.75, 95% CI 0.59–0.95); *p* = 0.017). The addition of Abiraterone improved the median OS from 4.72 years (SOC) to 5.72 years (SOC+abiraterone) (HR = 0.82, 95% CI 0.69–0.98; *p* = 0.030). Regarding patients with high tumor volume, there was a median survival benefit of 1.5 years in patients who were treated with the triplet (HR = 0.72, 95% CI 0.55–0.95; *p* = 0.019) [16].

As a result, triple therapies show a promising survival benefit in mHSPC patients, especially in those with high tumor burden. There are no comparisons between the triple and ADT + ARSI doublets available.

Table 3 provides a summary of the mentioned treatment options in mHSPC:

### 3.2. Metastatic Castration Resistant Prostate Cancer (mCRPC)

#### 3.2.1. Firstline Treatment in mCRPC

First-line treatment in men with mCRPC differ. Most trials explored the role of treatments after the failure of ADT alone. 

Docetaxel was reported to improve OS compared to Mitoxantrone in the SWOG 99–16 trial. Docetaxel/Estramustine (every 3 weeks 60 mg/m^2^) was compared to Mitoxantrone and prednisone (every 3 weeks 12 mg/m^2^). Patients who received docetaxel had an overall survival of 17.5 months whereas patients who were treated with Mitoxantrone had an OS of only 15.6 months (*p* = 0.02; HR = 0.80; 95% CI 0.67–0.97). There was a significant difference in rPFS:6.3 months (Docetaxel) vs. 3.2 months (Mitoxantrone) (*p* < 0.001) [17].

The efficacy of abiraterone acetate before chemotherapy was shown in the placebo controlled COU-AA-302 trial. Patients who received abiraterone acetate + prednisone had superior OS than those in placebo + prednisone group (34.7 vs. 30.3 months HR 0.81, *p* = 0.0033). There was also a significant improvement in rPFS favoring AAP (16.5 months vs. 8.3 months; *p* < 0.0001) [18].

PREVAIL investigated the therapeutic benefit of enzalutamide in comparison to placebo in mCRPC in chemotherapy-naïve patients. Patients treated with enzalutamide had an OS of 32.4 months compared to 30.2 months in placebo group (HR = 0.71, 95% CI 0.60–0.84, *p* < 0.001). rPFS was significantly longer in patients who received enzalutamide (20.0 months vs. 5.4 months (HR = 0.186, 95% CI 0.15–0.23; *p* < 0.0001) [19].

The PROPEL trial investigated the combination of Olaparib + Abiraterone vs. Placebo + Abiraterone. This trial will be mentioned in detail in section “Molecular Therapies”.

Firstline options in CRPC are summarized in Table 4. 

#### 3.2.2. Options after Pretreatment in mCRPC

For mCRPC patients who have already been treated with docetaxel, there are some therapeutic options, which will be listed below.

Cabazitaxel is a novel taxane with activity in docetaxel resistant CRPC. In the TROPIC 2013 trial, Cabazitaxel + prednisone showed a benefit in 2 year OS in comparison to mitoxantrone + prednisone (OS > 2 years in 15.9% (60/378) vs. 31/377 (8.2%). (odds ratio 2.11; 95% CI 1.33–3.33) [20].

The value of AAP in treatment of mHSPC has already been mentioned above. Additionally, in mCRPC it is a possible therapy option after docetaxel. In COU AA 301 study, AAP was compared to placebo/prednisone and led to an OS of 15.8 months in AAP group vs. 11.2 months in the placebo arm. (*p* < 0.0001, HR = 0.74, 95% CI: 0.64–0.86). Median rPFS was 5.6 months (5.6–6.5) in AAP group and 3.6 months (2.9–5.5) in control group (HR = 0.66, 0.58–0.76; *p* < 0·0001) [21].

Enzalutamide has been mentioned above. In the AFFIRM trial, patients who received enzalutamide after docetaxel had a significantly longer OS than those who received placebo (OS 18.4 vs. 13.6 months; (*p* < 0.001, HR = 0.63; 95% CI: 0.53–0.75)). There was also a statistically significant difference in rPFS: 8.3 months vs. 2.9 months (*p* < 0.001, HR = 0.63; 95% CI: 0.53–0.75) that favored enzalutamide [22].

In patients with two or more symptomatic bone metastases and no visceral metastases, alpharadin (Radium 223) is also a possible treatment option. Besides symptom relief in bone metastases, it also led to a longer OS in ALSYMPCA (Radium 223 vs. Placebo in previous or no previous docetaxel). OS in patients who received Radium 223 was 14.9 months vs. 11.3 months in placebo group (*p* = 0.002, HR = 0.61; 95% CI: 0.46–0.81) The most common hematologic AE in the Radium-223 group was anemia (187/600 pts.; 31%) whereas bone pain (300/600 pts.; 50%) and nausea (213/600 pts.; 36%) where most common nonhematologic AEs. [23].

In the CARD study, Cabazitaxel was compared to Abiraterone or Enzalutamide in patients with mCRPC who were treated with Doxetaxel and Abiraterone or Ezalutamide. In all, 255 patients were randomized in two groups: one group received Cabazitaxel (129 pts.), the other group received Abiraterone or Enzalutamide depending on which they had not received yet (126 pts.). The median overall survival was 13.6 months in the Cabazitaxel group and 11.0 months in the Abiraterone/Enzalutamide group (HR for death = 0.64; 95% CI, 0.46 to 0.89; *p* = 0.008). The median progression-free survival was 4.4 months in Cabazitaxel group vs. 2.7 months in the other group. (HR for progression or death =0.52; 95% CI, 0.40 to 0.68; *p* < 0.001). As a result, Cabazitaxel resulted in longer overall survival and progression-free survival [24].

Options after pretreatment are summarized in Table 5.

## 4. Molecular Therapy

The incorporation of molecular diagnostics deepened our understanding of putative therapeutic avenues in mCRPC. BRCA 1 or 2 regulate homologous recombination (HR) and loss in BRCA function may occur as germline or sporadic alteration. BRCA deficient cancers are explicitly susceptible to PARP inhibitors. The clinically most advanced PARP inhibitor in mCRPC is olaparib. The PROfound study compared olaparib with AAP or enzalutamide in patients with alterations in HR repair (HRR) deficient CRPC after treatment with a new hormonal agent. BRCA 1 alteration was found in 8/256 pts in Olaparib group (3%) and 5/131 pts. in control group (4%). BRCA 2 alteration was found in 81/256 pts (32%) in the Olaparib group and 47/131 (36%) in the control group. In patients with at least one alteration in BRCA1, BRCA 2 or ATM, Olaparib achieved a superior rPFS (7.39 vs. 3.55 mo. (HR = 0.34; 95% CI: 0.25–0.47; *p* < 0.0001)) and OS (18.5 mo vs. 15.1 mo. hazard ratio for death = 0.64; 95% CI, 0.43 to 0.97; *p* = 0.02). In the overall population, the median OS at interim analysis was 17.5 months (Olaparib group) and 14.3 months (control group) (hazard ratio for death = 0.67; 95% CI, 0.49 to 0.93). Patients with BRCA alterations derived the largest benefit, which led to a restricted label in Europe. However, other HRR alterations seem to select for PARPi-sensitivity, but subgroups remain small and their predictive strength remains vague [25].

In the PROpel study, 796 patients with mCRPC were randomized to receive Olaparib + Abiraterone (*n* = 399) vs. Placebo and Abiraterone (*n* = 397). In an interim analysis, rPFS was longer in Olaparib + Abiraterone group irrespective of HRR status (24.8 vs. 16.6 months; HR = 0.66, 95% confidence interval [CI] 0.54–0.81; *p* < 0.0001). Data on OS were immature at interim analysis [26].

Based on these data, BRCA testing has entered the clinical routine in mCRPC.

Alterations of the PI3 kinase/AKT signaling pathway are common in CRPC. PTEN is a suppressor of this signaling pathway and its loss is frequently found in CRPC. PTEN loss is associated with an aggressive clinical course and subject for pharmacological intervention in late stage mCRPC. Ipatasertib is an AKT inhibitor and its clinical efficacy was tested in a pivotal trial. Patients with previously untreated asymptomatic or mildly symptomatic mCRPC were eligible. The combination of Ipatasertib and AAP showed a benefit in rPFS in patients with PTEN loss in comparison to AAP + placebo (18.5 months vs. 16.5 months *p* = 0.0335, HR = 0.77, 95% CI: 0.61–0.98). There was no significant benefit on PFS in the intention to treat population (16.6 months (Placebo) vs. 19.2 months (HR = 0.84 [95% CI 0·71–0.99]; *p* = 0.043) [27].

A new therapeutic modality was introduced with the development of systemic PSMA-targeted radio ligand therapy (RLT) in mCRPC. Lutetium-177-PSMA-617 (LU-PSMA) is a small molecule that binds specifically to PSMA, which enables ß particle therapy to adjacent tumor cells in CRPC. A positive diagnostic 68-Gallium PSMA PET scan is a prerequisite to select suitable patients for this molecular therapy. 

The VISION trial tested LU-PSMA in previously treated mCRPC patients who were not candidates for chemotherapy. LU-PSMA showed a significant benefit in rPFS (8.7 vs. 3.4 mo. (*p* < 0.001; HR = 0.40; 99.2% CI: 0.29–0.57)) and OS (15.3 vs. 11.3 mo. (*p* < 0.001; HR = 0.62; 95% CI: 0.5–0.74)) in comparison to standard of care (best supportive care, which consisted of hormonal therapy, denosumab, bisphosphonates, radiation therapy or glucocorticoids) [28].

Another important trial which proved the effectiveness of PSMA radioligand therapy was TheraP. It included previously treated mCRPC patients who were deemed fit for chemotherapy and patients were randomized to receive LU-PSMA or Cabazitaxel. Patient selection was more stringent and permitted only patients with a match of PSMA- and FDG-PET activity. LU-PSMA RLT (up to six cycles; *n* = 98 pts.) was compared to Cabazitaxel (up to 10 cycles; *n* = 85 pts.). PSA response (defined as reduction of at least 50% from baseline), the primary endpoint, was shown in 66/98 (67%) pts who received PSMA RLT, but only 37/85 pts. (43%) who received Cabazitaxel (*p* < 0.001). Grade 3–4 adverse events occurred in 32 (33%) patients in the PSMA RLT group vs. 45 (53%) in the Cabazitaxel group. 

Overall, PSMA RLT showed improved efficacy and lower risk of grade 3–4 adverse events when compared to Cabazitaxel. However, the trial did not indicate major differences in OS in this trial and underlines the value of LU-PSMA in direct comparison to Cabazitaxel in mCRPC patients, which is considered a life-prolonging therapy [29].

Both BRCA 1/2 mutations and PSMA-positivity were predictive markers for treatment benefit. The molecular therapies are summarized in Table 6.

The most common adverse events related to molecular therapies are shown in Table 7. 

### 4.1. Immunotherapy

Until now, immunotherapy has not been of great importance in the treatment of prostate cancer and did not improve clinical outcomes significantly. Current trials test the role of ICI in specific subgroups: KEYNOTE- 641 is a Phase 3, randomized, double-blind, placebo-controlled trial, which investigates Pembrolizumab/Placebo in combination with Enzalutamide in mCRPC patients who were not treated with abiraterone or progressed on Abiraterone and did not receive chemotherapy [30]. Keynote-921 is a Phase 3, randomized, double-blind, placebo-controlled trial which investigates pembrolizumab/placebo in combination with docetaxel and prednisone in mCRPC patients who have received novel hormonal agents but no chemotherapy before [31]. On 3 August 2022, the sponsor announced that the study did not meet its primary endpoints (improvement in overall survival and rPFS). KEYNOTE-991 is a Phase 3 trial to investigate the role of Pembrolizumab in combination with Enzalutamide and ADT versus Placebo in combination with Enzalutamide and ADT in mCRPC patients who did not receive novel hormonal agents before. The results of these trials are not available until 2025/2026 and could reform the rule of immunotherapy in prostate cancer [32]. Keynote-365 is a multicohort phase Ib/II study which tests pembrolizumab in combination with Olaparib (Cohort A), Docetaxel and Prednisone (Cohort B), Enzalutamide (Cohort C). In the results published so far, PSA response was reported in 9% of patients in cohort A, 28% in cohort B and 22% in cohort C. The final results of this study are not yet available [33].

### 4.2. Cell Based Immunotherapy

So far, cell based immunotherapy has not found its place in the treatment landscape of mCRPC. This could change in the future due to ongoing developments.

A therapy approach which was developed several years ago is supileucel-T. This is an active cell based autologous immunotherapy. Peripheral blood mononuclear cells are activated ex vivo with PA2024, a recombinant fusion protein of prostate antigen, which causes immune cell activation. Ultimately, mCRPC patients with an expected survival of at least 6 months received supileucel-T in a phase III trial (*n* = 341 pts.). In all, 171 patients received placebo as a control group. The OS in the supileucel-T group was 25.8 months and 21.7 months in the placebo group. (HR for death in the sipuleucel-T group = 0.78; 95% confidence interval [CI], 0.61 to 0.98; *p* = 0.03) [34]. Although showing principal activity, supileucel-T is not available in routine practice anymore.

## 5. Vaccination

Vaccination against prostate cancer is not established in clinical practice. Nevertheless, there are studies that provided promising data. As an example, PROSTVAC, a PSA recombinant vaccinia vector, showed promising activity in mCRPC patients. Median OS was 8.5 months longer in pts who received PROSTVAC than in the control group (*p* = 0.0061) [35]. However, confirmatory trials were negative and did not show a benefit in OS [36].

## 6. Bispecific T-Cell Engager

The most advanced bispecific T-cell engager in the therapy of prostate cancer is PSMA Bite. PSMA is expressed in prostate cancer cells and metastases and can be used to specifically target therapies to prostate cancer cells, such as for LU-PSMA radioligand therapy [28].

PSMA Bite is a new experimental treatment for mCRPC patients. PSMA Bite is a bispecific CD3 and PSMA antibody construct, which re-directs and activates T-cells to PSMA expressing cells.

Bispecific T-cell engager (BiTE) is already established in the treatment of other malignancies. Blinatumumab was the first approved BITE therapy. It is a bispecific monoclonal antibody construct that causes CD3 positive T-cells to recognize and target CD 19 positive B-cells. Blinatumumab is approved for patients with refractory or relapsed precursor B-ALL. Compared to chemotherapy, blinatumumab showed a survival benefit in patients with pretreated B-ALL: Median OS in the blinatumomab group was 7.7 months compared to 4.0 months in the chemotherapy group (HR = 0.71; 95% CI 0.55 to 0.93; *p* = 0.01) [37].

Based on promising data from the treatment of hematological diseases, the suitability of bite molecules for the treatment of solid tumors is currently being investigated.

A Phase I study of pasotuximab (PSMA Bite) tested its tolerability and activity in mCRPC. It included 68 patients in two cohorts of subcutaneous (s.c.) or intravenous (i.v.) application. These patients were pretreated with at least one taxane regimen and refractory to AAP or enzalutamide. Next, to evaluate the maximum tolerated dose in both cohorts, PSA response was investigated. In the subcutaneous cohort, every patient developed anti-drug antibodies, which led to the premature discontinuation of the s.c. application cohort. 

PSA decline was −24.7% in pts. with s.c. treatment. In the i.v. group, median best PSA change was −22.0, −37.7 and −54.9% in 20, 40 and 80 µg/d dose cohorts. One patient in i.v. cohort had <50% PSA reduction for 50 weeks and stable disease for 337 days. Another patient in the i.v. cohort had nearly complete regression of lymphnode and bone metastases in PSMA-PET CT. PSA progression in long-term responders in i.v. cohort occurred after 11–17 months and indicates the principle and dose-dependent activity of this new modality. Most common adverse events in both cohorts were fever (81% in s.c. and 94% in i.v. cohort), injection site reaction in the s.c. cohort (24/31; 77%), chills (23% in s.c. and 69% in the i.v. cohort) and fatigue (36% in the s.c. and 31% in the i.v. cohort). Treatment-emergend AEs occurred in both cohorts. Most common were anemia (39%) in the s.c. cohort and decreased lymphocyte count (44%) and infections (31%) in the i.v. cohort [38]. Further trials to investigate the value and safety of PSMA Bite are ongoing.

Other targets than PSMA may be used for bispecific T-cell engager, such as Glypican-1 [39] and ADAM 17 (disintegrin and metalloproteinase 17) [40] or STEAP-1 [41], which are under investigation.

**Figure 1:**
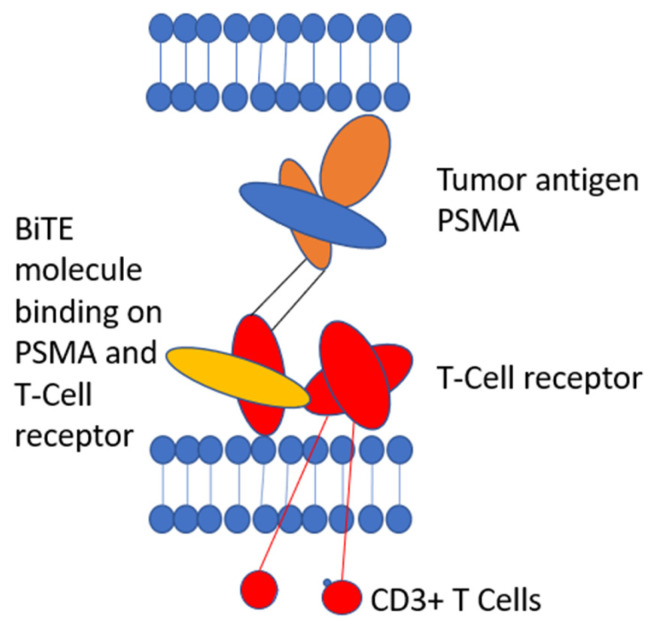
Bispecific T-cell engager. Modified according to Strohl et al. [42].

Overall, bispecific T-cell engager (Figure 1) is a promising new therapy option with early signs of clinical activity. Of course, further clinical studies are necessary before they are ready for prime time, but early clinical trials are promising.

## 7. Conclusions

Prostate cancer is the most common solid cancer in men. In recent years, there have been major advances in the treatment of metastatic prostate cancer, which have pushed frontiers of survival expectations to new levels. ADT alone is not enough anymore for men with metastatic HSPC. Men with BRCA 1/2 alterations and with PSMA-positive cancers benefit from targeted treatment with PARPi or Lu-PSMA, respectively. However, castration resistant prostate cancer remains a deadly disease and new therapies are needed. The advent of molecular therapies, such as RLT or PARPi, advanced the field more recently and early clinical trials indicate promising new therapeutic approaches, which includes immunotherapies.

## Figures and Tables

**Table 1:** CHAARTED criteria [5].

High Volume	Low Volume
At least 4 bone metastases - Including at least 1 outside vertebral column or pelvis or visceral metastasis	High volume criteria not met

**Table 2:** LATITUDE Criteria [6].

High Risk	Low Risk
At least 2 risk factors of: - At least 3 bone metastasis - Visceral metastasis - ISUP 4 or higher	High risk criteria not met

**Table 3:** List of treatment options and survival rates in mHSPC.

Therapy	Trial Name	No. of Pts	Key Findings	Statistics
ADT	STAMPEDE (control arm)	917	Median OS: 43.1 months	IQR (22.7–90.7 months)
ADT + Docetaxel	STAMPEDE	SOC (ADT): 1184 pts. Docetaxel: 592 pts.	43.1 months (SOC) 59.1 months (ADT + Docetaxel)	HR = 0.81 *p* = 0.003
ADT + Docetaxel	CHAARTED	SOC (ADT): 393 Docetaxel 397	SOC: 47.2 months ADT + Docetaxel 57.6 months	HR = 0.72 *p* = 0.0018
ADT + AAP	STAMPEDE	SOC (ADT): 957 ADT + AAP: 960	3 year OS: SOC: 76% ADT + AAP: 83%	HR = 0.63 *p* < 0.001
ADT + AAP	LATITUDE	ADT + Placebo: 597 ADT: AAP 602	3 year OS: SOC: 49% ADT + AAP: 66%	HR = 0.62 *p* < 0.001
ADT + Enzalutamide	ENZAMET	ADT + Bicalutamide/Nilutamide/Flutamide (SOC): 562 ADT: Enzalutamide 563	3 year OS: SOC: 72% ADT + Enzalutamide: 80%	HR = 0.67 *p* = 0.002
ADT + Apalutamide	TITAN	ADT + Placebo: 527 ADT + Apalutamide: 525	24 months OS: ADT + Placebo: 73.5% ADT + Apalutamide: 82.4%	HR = 0.67 *p* = 0.005
Darolutamide + ADT + Docetaxel	ARASENS	Darolutamide + ADT + Docetaxel: 651 Darolutamide + ADT + Placebo: 655	Risk of death 32.5% lower in Darolutamide group	HR = 0.68 *p* < 0.001
Docetaxel + Abiraterone acetate/Prednisone + ADT	PEACE-1	SOC: ADT 118; ADT + Docetaxel 178 ADT + Docetaxel + Abiraterone: 177 ADT + Abiraterone: 115	Median OS 4.72 years in SOC vs. 5.72 years (SOC + Abiraterone) 25% reduction in the risk of death AAP + ADT+ Docetaxel in pts. with high tumor burden: survial benefit of 1.5 years	HR = 0.82 *p* = 0.030 HR = 0.75 *p* = 0.017 HR = 0.72 *p* = 0.019

**Table 4:** Firstline options in CRPC.

Therapy	Trial Name	Key Findings	Statistics
Docetaxel/Estramustine vs. Mitoxantrone	SWOG 99–16	17.5 mo (Docetaxel) vs. 15.6 mo. (Mitoxantrone)	*p* = 0.02 HR = 0.80
Abiraterone+ Prendisolone vs. Prednisolone + Placebo	COU-AA-302	34.7 (AAP) vs. 30.2 months (Prednisolone + Placebo)	*p* = 0.0033 HR = 0.81
Enzalutamide vs. Placebo	PREVAIL	OS 32.4 (Enzalutamide) vs. 30.2 mo	*p* < 0.001

**Table 5:** Options after pretreatment in mCRPC.

Treatment	Pre-Therapy, Main Inclusion Criteria	Trial	Main Result	Statistics
Cabazitaxel + Prednisolone vs. Mitoxantrone + Prednisolone	Docetaxel	TROPIC 2013	2 year OS 15.9% (Cabazitaxel) vs. 8.2% (Mitoxantrone).	Odds ratio 2.11 95% CI 1.33–3.33
Abiraterone Acetate + Predni vs. Placebo + Predni	Docetaxel	COUAA301	OS 15.8 (Abiraterone) vs. 11.2 mo.	*p* < 0.0001 HR = 0.74
Enzalutamide vs. Placebo	Docetaxel	AFFIRM 2012	OS 18.4 (Enzalutamide) vs. 13.6 mo.	*p* < 0.001 HR = 0.63
Cabazitaxel vs. Abiraterone or Enzalutamide	Docetaxel and Abiraterone or Enzalutamide	CARD	OS 13.6 (Cabazitaxel) vs. 11.0 mo.	*p* = 0.008 HR = 0.64
Radium 223 vs. Placebo	Previous or no previous docetaxel Two or more symptomatic bone mts. No visceral mts.	ALSYMPCA 2013	OS 14.9 (Radium 223) vs. 11.3 mo.	*p* = 0.002 HR = 0.61

**Table 6:** Molecular therapies.

Treatment	Pre-Therapy, Main Inclusion Criteria	Trial	Main Result	Statistics
Olaparib vs. AAP or Enzalutamide	pts. with alterations in HRR mutated genes after at least 1 ADT	PROfound	OS 19.1 vs. 14.7 mo. (in pts. with BRCA 1/2, Atm alterations) rPFS 7.39 vs. 3.55 mo.	*p* = 0.0175 HR = 0.69 *p* < 0.001 HR = 0.34
Ipatasertib + AAP vs. Placebo + AAP	Pts. with untreated CRPC	Ipatential 150	rPFS in PTEN loss group 18.5 vs. 16.5 mo.	*p* = 0.0355 HR = 0.77
PSMA RLT LU-PSMA vs. LU-PSMA + SOC or SOC alone	Pretreatment with ADT and taxane regimen	VISION	PFS 8.7 vs. 3.4 mo.	*p* < 0.001 HR = 0.62
PSMA RLT vs. Cabazitaxel	mCRPC, Cabazitaxel next appropriate treatment	TheraP	PSA response: 66/98 (PSMA RLT) pts. vs. 37/85 pts.	*p* < 0.001

**Table 7:** Most common adverse events related to molecular therapy.

Treatment	Trial	Most Common AE
Olaparib	ProFound	Anemia (119/256) = 46% Nausea (106/256) = 41% Fatigue or asthenia (105/256) = 41%
Ipatasertib	Ipatential150	Diarrhoea (440/551) = 80% Hypergylcaemia (264/551) = 48% Rash (228/256) = 41%
PSMA RLT	Vision	Fatigue 228/519 = 43.1% Dry mouth 205/519 = 38.8% Nausea 187/519 = 35.3% Anemia 168/519 = 31.8%

## References

[1] Siegel R.L., Miller K.D., Fuchs H.E., Jemal A. (2022). Cancer statistics, 2022. CA Cancer J. Clin..

[2] Yu E.Y., Klaasen Z. ESM0 2022: Pembrolizumab Plus Olaparib vs. Abiraterone or Enzalutamide for Patients with Previously Treated mCRPC: Randomized Open-Label Phase 3 Keylink 010 Study. https://www.urotoday.com/conference-highlights/esmo-2022/esmo-2022-prostate-cancer/139443-esmo-2022-pembrolizumab-olaparib-versus-abiraterone-or-enzalutamide-for-patients-with-previously-treated-mcrpc-randomized-open-label-phase-3-keylynk-010-study.html.

[3] Slovin S.F., Higano C.S., Hamid O., Tejwani S., Harzstark A., Alumkal J.J., Scher H.I., Chin K., Gagnier P., Mc Henry M.B. (2013). Ipilimumab alone or in combination with radiotherapy in metastatic castration-resistant prostate cancer: Results from an open-label, multicenter phase I/II study. Ann. Oncol..

[4] Tucker M.D., Zhu J., Marin D., Gupta R.T., Gupta S., Berry W., Ramalingam S., Zhang T., Harrison M., WU Y. (2019). Pembrolizumab in men with heavily treated metastatic castrate-resistant prostate cancer. Cancer Med..

[5] Gravis G., Boher J.M., Chen Y.H., Liu G., Fizazi K., Carducci M.A., Oudard S., Joly F., Jarrard D.M., Soulie M. (2018). Burden of Metastatic Castrate Naive Prostate Cancer Patients, to Identify Men More Likely to Benefit from Early Docetaxel: Further Analyses of CHAARTED and GETUG-AFU15 Studies. Eur. Urol..

[6] Fizazi K., Tran N., Fein L., Matsubara N., Rodriguez-Antolin A., Alekseev B.Y., Özgüroglu M., Ye D., Feyerabend S., Protheroe A. (2017). Abiraterone plus Prednisone in Metastatic, Castration-Sensitive Prostate Cancer. N. Engl. J. Med..

[7] Francini E., Gray K.P., Xie W., Shaw G.K., Valnca L., Bernard B., Albiges L., Harshman L.C., Lantoff P.W., Palin M.E. (2018). Time of metastatic disease presentation and volume of disease are prognostic for metastatic hormone sensitive prostate cancer (mHSPC). Prostate.

[8] James N.D., Spears M.R., Clarke N.W., Dearnaley D.P., De Bono J.S., Gale J., Hetherington J., Hoskin P.J., Jones R.J., Laing R. (2015). Survival with Newly Diagnosed Metastatic Prostate Cancer in the ‘Docetaxel Era’: Data from 917 Patients in the Control Arm of the STAMPEDE Trial (MRC PR08, CRUK/06/019). Eur. Urol..

[9] Clarke N.W., Ali A., Ingleby F.C., Hoyle A., Amos C.L., Attard G., Brawley C.D., Calvert J., Chowdhury S., Cook A. (2019). Addition of docetaxel to hormonal therapy in low- and high-burden metastatic hormone sensitive prostate cancer: Long-term survival results from the STAMPEDE trial. Ann. Oncol..

[10] Kyriakopoulos C.E., Chen Y.-H., Carducci M.A., Liu G., Jarrard D.F., Hahn N.M., Shevrin D.H., Dreicer R., Hussain M., Dreicer R. (2018). Chemohormonal Therapy in Metastatic Hormone-Sensitive Prostate Cancer: Long-Term Survival Analysis of the Randomized Phase III E3805 CHAARTED Trial. J. Clin. Oncol..

[11] James N.D., De Bono J.S., Spears M.R., Clarke N.W., Mason M.D., Dearnaley D.P., Ritchie A., Amos C.L., Gilson C., Jones R.J. (2017). Abiraterone for Prostate Cancer Not Previously Treated with Hormone Therapy. N. Engl. J. Med..

[12] Davis I.D., Martin A.J., Stockler M.R., Begbie S., Chi K.N., Chowdhury S., Coskinas X., Frydenberg M., Hague W.E., Horvath L.G. (2019). Enzalutamide with Standard First-Line Therapy in Metastatic Prostate Cancer. N. Engl. J. Med..

[13] Chi K.N., Agarwal N., Bjartell A., Chung B.H., Gomes A., Given R., Soto A.J., Merseburger A.S., Özgüroglu M., Uemura H. (2019). Apalutamide for Metastatic, Castration-Sensitive Prostate Cancer. N. Engl. J. Med..

[14] Chi K.N., Chowdhury S., Bjartell A., Chung B.H., Gomes A., Given R., Juarez A., Merseburger A., Özguroglu M., Uemura H. (2021). Apalutamide in Patients With Metastatic Castration-Sensitive Prostate Cancer: Final Survival Analysis of the Randomized, Double-Blind, Phase III TITAN Study. J. Clin. Oncol..

[15] Smith M.R., Hussain M., Saad F., Fizazi K., Sternberg C.N., Crawford E.D., Kopyltsov E., Park C.H., Alekseev B., Montesa-Pino A. (2022). Darolutamide and Survival in Metastatic, Hormone-Sensitive Prostate Cancer. N. Engl. J. Med..

[16] Fizazi K., Galceran J.C., Foulon S., Roubaud G., McDermott R., Flechon A., Tombal B., Supiot S., Bertold D.R., Ronchin P. (2021). LBA5 A phase III trial with a 2×2 factorial design in men with de novo metastatic castration-sensitive prostate cancer: Overall survival with abiraterone acetate plus prednisone in PEACE-1. Ann. Oncol..

[17] Petrylak D.P., Tangen C.M., Hussain M.H.A., Lara P.N., Jones J.A., Taplin M.E., Burch P.A., Berry D., Moinpur C., Kohli M. (2004). Docetaxel and Estramustine Compared with Mitoxantrone and Prednisone for Advanced Refractory Prostate Cancer. N. Engl. J. Med..

[18] Ryan C.J., Smith M.R., de Bono J.S., Molina A., Logothetis C.J., de Souza P., Fizazi K., Mainwaring P., Piulats J.M., Ng S. (2013). Abiraterone in Metastatic Prostate Cancer without Previous Chemotherapy. N. Engl. J. Med..

[19] Beer T.M., Armstrong A.J., Rathkopf D.A., Loriot Y., Sternberg C.N., Higano C.S., Iversen P., Bhattcharia S., Carles J., Chowdhury S. (2014). Enzalutamide in Metastatic Prostate Cancer before Chemotherapy. N. Engl. J. Med..

[20] Bahl A., Oudard S., Tombal B., Özguroglu M., Hansen S., Kocak I., Gravis D., Devin J., Shen L., de Bono J.S. (2013). Impact of cabazitaxel on 2-year survival and palliation of tumour-related pain in men with metastatic castration-resistant prostate cancer treated in the TROPIC trial. Ann. Oncol..

[21] Fizazi K., Scher H.I., Molina A., Logothetis C.J., Chi K.N., Jonas R.J., Staffurth J.N., North S., Vogelzang S., Saad F. (2012). Abiraterone acetate for treatment of metastatic castration-resistant prostate cancer: Final overall survival analysis of the COU-AA-301 randomised, double-blind, placebo-controlled phase 3 study. Lancet Oncol..

[22] Scher H.I., Fizazi K., Saad F., Taplin M.E., Sternberg C.N., Miller K., de Wit R., Mulders P., Chi K.N., Shore N.D. (2012). Increased Survival with Enzalutamide in Prostate Cancer after Chemotherapy. N. Engl. J. Med..

[23] Parker C., Nilsson S., Heinrich D., Helle S.I., O’Sullivan J.M., Fossa S.D., Chodacki A., Wiechno P., Logue J., Seke M. (2013). Alpha Emitter Radium-223 and Survival in Metastatic Prostate Cancer. N. Engl. J. Med..

[24] de Wit R., de Bono J., Sternberg C.N., Fizazi K., Tombal B., Wülfing C., Kramer G., Eymard J.C., Bamias A., Calers J. (2019). Cabazitaxel versus Abiraterone or Enzalutamide in Metastatic Prostate Cancer. N. Engl. J. Med..

[25] de Bono J., Mateo J., Fizazi K., Saad F., Shore N., Sandhu S., Chi K.M., Sartor O., Agarwal N., Olmos D. (2020). Olaparib for Metastatic Castration-Resistant Prostate Cancer. N. Engl. J. Med..

[26] Saad F., Armstrong A.J., Thiery-Vuillemin A., Oya M., Loredo E., Procopio G., Menezes J., Calgiovanni Girotto G., Arslan C., Mehra N. (2022). PROpel: Phase III trial of olaparib (ola) and abiraterone (abi) versus placebo (pbo) and abi as first-line (1L) therapy for patients (pts) with metastatic castration-resistant prostate cancer (mCRPC). J. Clin. Oncol..

[27] Sweeney C., Bracarda S., Sternberg C.N., Chi K.N., Olmos D., Sandhu S., Massard C., Matsubara N., Alekseev B., Parnis F. (2021). Ipatasertib plus abiraterone and prednisolone in metastatic castration-resistant prostate cancer (IPATential150): A multicentre, randomised, double-blind, phase 3 trial. Lancet.

[28] Sartor O., de Bono J., Chi K.N., Herrmann K., Rahbar K., Tagawa S.T., Nordquist L.T., Vaishampajan N., El-Haddad G., Park C.H. (2021). Lutetium-177–PSMA-617 for Metastatic Castration-Resistant Prostate Cancer. N. Engl. J. Med..

[29] Hofman M.S., Emmet L., Sandhu S., Iravani A., Joshua A.M., Goh J.C., Pattison D.A., Tan T.H., Kirkwood I.D., Ng S. (2021). [177Lu]Lu-PSMA-617 versus cabazitaxel in patients with metastatic castration-resistant prostate cancer (TheraP): A randomised, open-label, phase 2 trial. Lancet.

[30] Graff J.N., Liang L.W., Kim J., Stenzl A. (2021). KEYNOTE-641: A Phase III study of pembrolizumab plus enzalutamide for metastatic castration-resistant prostate cancer. Future Oncol..

[31] Petrylak D.P., Ratta R., Gafanov R., Facchini G., Piulats J.M., Kramer G., FLaig T.W., Chandana S.R., Li B., Burgents J. (2021). KEYNOTE-921: Phase III study of pembrolizumab plus docetaxel for metastatic castration-resistant prostate cancer. Future Oncol..

[32] Gratzke C., Niu C., Poehlein C., Burgents J. (2020). 346 KEYNOTE-991: Phase 3 study of pembrolizumab plus enzalutamide and androgen deprivation therapy (ADT) for patients with metastatic hormone-sensitive prostate cancer (mHSPC). J. Immunother. Cancer.

[33] Joshua A.M., Gurney H., Retz M., Tafreshi A., Fong P.C.C., Shore N.D., Romano E., Augustin M., Piulats J.M., Berry W.R. (2020). Pembrolizumab (pembro) Combination Therapies in Patients With Metastatic Castration-Resistant Prostate Cancer (mCRPC): Cohorts A-C of the Phase 1b/2 KEYNOTE-365 Study. Ann. Oncol..

[34] Kantoff P.W., Higano C.S., Shore N.D., Berger E.R., Small E.J., Penson D.E., Redfern C.H., Ferrari A.C., Dreicer R., Sims R.B. (2010). Sipuleucel-T Immunotherapy for Castration-Resistant Prostate Cancer. N. Engl. J. Med..

[35] Kantoff P.W., Schuetz J., Blumenstein B.A., Glode L.M., Bilhartz D.L., Wyand M., Manson K., Panicali D.L., Laus R., Schlom J. (2010). Overall Survival Analysis of a Phase II Randomized Controlled Trial of a Poxviral-Based PSA-Targeted Immunotherapy in Metastatic Castration-Resistant Prostate Cancer. J. Clin. Oncol..

[36] Gulley J.L., Borre M., Vogelzang N., Ng S., Agarwal N., Parker C.C., Pook D.W., Rathenborg P., Flaig T., Carles J. (2019). Phase III Trial of PROSTVAC in Asymptomatic or Minimally Symptomatic Metastatic Castration-Resistant Prostate Cancer. J. Clin. Oncol..

[37] Kantarjian H., Stein A., Gökbuket N., Fiedling A.K., Schuh A.C., Ribera J.M., Wei A., Dombret H., Foa R., Bassan R. (2017). Blinatumomab versus Chemotherapy for Advanced Acute Lymphoblastic Leukemia. N. Engl. J. Med..

[38] Hummel H.-D., Kufer P., Grüllich C., Seggewiss-Bernhardt R., Deschler-Baier B., Chatterjee M., Goebeler M.E., Miller K., de Santis M., Loidl W. (2021). Pasotuxizumab, a BiTE ^®^ immune therapy for castration-resistant prostate cancer: Phase I, dose-escalation study findings. Immunotherapy.

[39] Lund M.E., Howard C.B., Thurecht K.J., Campbell D.H., Mahler S.M., Walsh B.J. (2020). A bispecific T cell engager targeting Glypican-1 redirects T cell cytolytic activity to kill prostate cancer cells. BMC Cancer.

[40] Yamamoto K., Trad A., Baumgart A., Hüske L., Lorenzen I., Chalaris A., Grötzinger J., Dechow T., Scheller J., Rose-John S. (2012). A novel bispecific single-chain antibody for ADAM17 and CD3 induces T-cell-mediated lysis of prostate cancer cells. Biochem. J..

[41] Lin T.-Y., Park J.A., Long A., Guo H.-F., Cheung N.-K. (2021). Novel potent anti-STEAP1 bispecific antibody to redirect T cells for cancer immunotherapy. J. Immunother. Cancer.

[42] Strohl W.R., Naso M. (2019). Bispecific T-Cell Redirection versus Chimeric Antigen Receptor (CAR)-T Cells as Approaches to Kill Cancer Cells. Antibodies.

